# The Prevalence of Heterozygous Familial Hypercholesterolemia in Selected Regions of the Russian Federation: The FH-ESSE-RF Study

**DOI:** 10.3390/jpm11060464

**Published:** 2021-05-24

**Authors:** Alexey N. Meshkov, Alexandra I. Ershova, Anna V. Kiseleva, Svetlana A. Shalnova, Oxana M. Drapkina, Sergey A. Boytsov, on behalf of the FH-ESSE-RF Investigators

**Affiliations:** 1 Federal State Institution, National Medical Research Center for Therapy and Preventive Medicine, Min-istry of Healthcare of the Russian Federation, Petroverigsky per., 10, bld. 3, 101000 Moscow, Russia; alersh@mail.ru (A.I.E.); sanyutabe@gmail.com (A.V.K.); SShalnova@gnicpm.ru (S.A.S.); drapkina@bk.ru (O.M.D.); 2 National Medical Research Center for Cardiology, 3-ya Cherepkovskaya Street, 15A, 121552 Moscow, Russia; prof.boytsov@gmail.com

**Keywords:** familial hypercholesterolemia, Russia, prevalence, coronary artery disease, *LDLR*, *APOB*, *PCSK9*

## Abstract

Heterozygous familial hypercholesterolemia (HeFH) is one of the most common genetic conditions but remains substantially underdiagnosed. The aim of our study was to investigate the prevalence of HeFH in the population of 11 different regions of Russia. Individuals were selected from the Epidemiology of Cardiovascular Risk Factors and Diseases in Regions of the Russian Federation Study. All participants who had low-density lipoprotein cholesterol (LDL-C) higher than 4.9 mmol/L, or LDL-C lower than 4.9 mmol/L, but had statin therapy, were additionally examined by FH experts. FH was diagnosed using the Dutch Lipid Clinic Network criteria, incorporating genetic testing. HeFH prevalence was assessed for 18,142 participants. The prevalence of patients with definite or probable HeFH combined was 0.58% (1 in 173). A total of 16.1% of patients with definite or probable HeFH had tendon xanthomas; 36.2% had mutations in one of the three genes; 45.6% of FH patients had coronary artery disease; 63% of HeFH patients received statins; one patient received an additional PCSK9 inhibitor; no patients received ezetimibe. Only 3% of patients reached the LDL-C goal based on 2019 ESC/EAS guidelines. Underdiagnosis and undertreatment of FH in Russia underline the need for the intensification of FH detection with early and aggressive cholesterol-lowering treatment.

## 1. Introduction

Heterozygous familial hypercholesterolemia (HeFH) is an autosomal dominant disorder known to be associated with elevated cholesterol levels and increased risk of premature coronary artery disease (CAD). Historically, the community prevalence of HeFH is estimated to be 1 in 500 [1]. Recent data suggest that the real prevalence of HeFH is underestimated [2]. The Copenhagen General Population Study (CGP Study) was the first unselected, community-based population study that assessed the prevalence of HeFH. The prevalence of individuals classified with definite or probable FH combined was 1 in 223 [3,4]. Reanalysis of survey data CGP Study in 2018 showed prevalence of HeFH as 1 in 218 [5]. In two meta-analyses of 2020, similar results were obtained on the prevalence of HeFH in the general population, 1/311 and 1/313, and it was shown that it varies by geographical location [6,7]. Previously, we showed that the prevalence of HeFH in the two West Siberian regions of the Russian Federation is 1 in 108 [8].

Due to the lifelong exposure to elevated levels of low-density lipoprotein cholesterol (LDL-C), an early pharmacological hypolipidemic treatment is the best approach to reduce the risk of premature cardiovascular (CV) events and CAD mortality in FH patients. In FH patients at very high risk of atherosclerotic cardiovascular disease (ASCVD) due to a prior history of ASCVD or another major risk factor, LDL-C goals are a >50% reduction of LDL-C from baseline and a concentration of LDL-C < 1.4 mmol/L. In the absence of ASCVD or another major risk factor, patients with FH are categorized as high risk, and LDL-C goals are a >50% reduction of LDL-C from baseline and a concentration of LDL-C < 1.8 mmol/L [9].

The aim of our study is to investigate the prevalence of HeFH in the population of the different regions of the Russian Federation and then to estimate the frequency of CAD and treatment with cholesterol-lowering medication in HeFH patients.

## 2. Materials and Methods

### 2.1. Sampling and Clinical Examination

**Figure 1:**
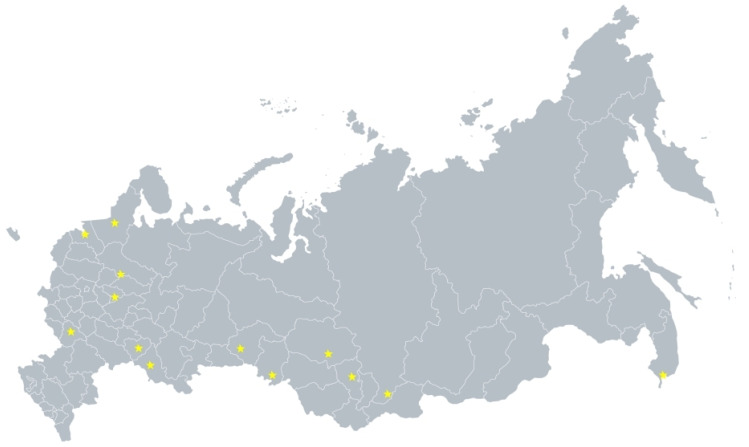
The location of the regions from the FH-ESSE-RF study. Yellow symbols indicate the regions from the FH-ESSE-RF study.

The FH-ESSE-RF study is a cross-sectional, non-interventional, multicenter study aimed at identifying HeFH in the population of the different regions of the Russian Federation. Participants for our study were selected from the Epidemiology of Cardiovascular Risk Factors and Diseases in Regions of the Russian Federation Study (ESSE-RF Study). The ESSE-RF is a study of a general population initiated in 2012 and covering 13 regions of Russia differing in climatic, geographic, economic, and demographic characteristics [8,10] (Figure 1). These regions are representative for the monitoring of cardiovascular health of the Russian population. A total of 21,300 participants were included in the study (about 1600 people aged 25–64 years from every region). Individuals were selected using cluster sampling. Data were obtained from questionnaires administered face-to-face, by a brief physical examination, and nonfasting venous blood samples. The level of LDL-C was measured directly in all participants. All subjects were interviewed to assess statin treatment. It should be emphasized that the ESSE-RF was not built for examination of FH and did not include the information about family history, xanthoma, and DNA testing, but it provided epidemiological data about participants that were used for the objectives of this study. The sample of this study included participants of the ESSE-RF conducted in the 13 regions (Table 1) who had LDL-C higher than 4.9 mmol/L, or who had LDL-C in the range of 1.8 to 4.9 mmol/L during treatment with statins. These eligible subjects were invited for examination and interviewed by experts in FH in the FH-ESSE-RF study. The following characteristics were recorded on the visit: age, sex, history of CAD, ischemic stroke, cerebral or peripheral vascular disease, history of lipid levels, lipid-lowering therapy status, family history of dyslipidemia, and cardiovascular diseases (CVD). On the visit, blood samples were taken for biobanking, lipid measurement, exclusion of secondary forms of hypercholesterolemia, and for genetic testing. The initial characteristics of the study participants, selection criteria, and examination methods were described in detail earlier [8,11]. In six regions, the levels of Lp(a) and ApoB were additionally determined as described earlier [12].

The diagnosis of FH was determined using the Dutch Lipid Clinic Network (DLCN) criteria, incorporating genetic testing [13]:

- Family history of premature CAD (<55 years for men; <60 years for women) in a first-degree relative and/or an increase of LDL-C more than 4.9 mmol/L in first-degree relatives (1 point) or first-degree relative with tendon xanthoma and/or corneal arcus and/or child(ren) < 18 years with LDL-C more than 3.9 mmol/L (2 points);
- Clinical history of premature CAD (ages as above, 2 points) or premature cerebral or peripheral vascular disease (ages as above, 1 point) in the subject;
- Presence of tendon xanthomata (6 points) or presence of corneal arcus in the subject under the age of 45 (4 points);
- Level of LDL-C in the subject higher than 8.5 mmol/L (>325 mg/dL) (8 points), 6.45–8.5 mmol/L (251–325 mg/dL) (5 points), 4.91–6.44 mmol/L (191–250 mg/dL) (3 points), or 4.0–4.9 mmol/L (155–190 mg/dL) (1 point);
- Causative mutation detected in the *LDLR*, *APOB*, or *PCSK9* genes (8 points).

The genetic test was performed on all participants with a clinical diagnosis of definite or probable HeFH and on all participants with LDL-C level 6.45 mmol/L and more in all regions except Ivanovo, where the genetic test was performed on all 1883 participants of the ESSE-RF study. CAD and cerebral and peripheral vascular diseases were established on the basis of data provided by medical documentation brought by the participant on the visit. Data about relatives were collected from medical records brought by the participant on the visit or orally obtained. A diagnosis of HeFH was considered definite if the total score was greater than 8, probable if the score was 6–8, possible if the score was 3–5, and unlikely if the score was below 3 points. All data were collected in a specially developed web registration form and stored on a protected server. The study was approved by the Independent Ethics Committee of the National Medical Research Center for Therapy and Preventive Medicine (protocol number 07-03/12 from 03.07.2012 and protocol number 04-04/17 from 06.06.2017) and conducted in accordance with the Helsinki Declaration. Informed written consent was obtained from each participant.

### 2.2. Genetic Analysis

The whole blood with EDTA from the participants collected at the last visit or blood from the biobank obtained as part of the ESSE-RF study were used for genetic testing. DNA was isolated using the QIAamp DNA Blood Mini Kit (Qiagen, Hilden, Germany). DNA concentration was determined on Qubit 4.0 fluorimeter (Thermo Fisher Scientific, Waltham, MA, USA). The next generation sequencing (NGS) was carried out on Ion S5 (Thermo Fisher Scientific, Waltham, MA, USA) for all participants except for participants from Ivanovo region. Ampliseq libraries were prepared on Ion Chef System (Thermo Fisher Scientific, Waltham, MA, USA) using a custom panel developed in the Ion AmpliSeq Designer software v7.4.2 (Thermo Fisher Scientific, Waltham, MA, USA). The panel included exonic and adjacent intronic sequences of 25 genes (UTR + CDS + 100 bp padding) for which, according to literature data, an association with hereditary dyslipidemias including *LDLR*, *APOB*, and *PCSK9* was found. VCF files were generated from BAM files on Torrent Server (Thermo Fisher Scientific, Waltham, MA, USA) with default parameters. VCF files were annotated using Ion Reporter v5.18.0.1 (Thermo Fisher Scientific, Waltham, MA, USA) with Annotate Variants analysis tool. For participants from Ivanovo region, NGS was carried out on Nextseq 550 (Illumina, San Diego, CA, USA). The library preparation was performed using the SeqCap EZ Prime Choice Library kit (Roche, Basel, Switzerland). The Roche panel was used, consisting of 244 (CDS + 25 bp padding) genes including *LDLR*, *APOB*, and *PCSK9*. Reads were aligned to the reference genome (GRCh37). Sequencing analysis resulted in fastq files. Data processing was performed with BWA, Picard, bcftools, GATK3 and generally followed the GATK best practices for variant calling. We applied standard GATK hard filters for single nucleotide substitutions (MQ, QD, FS, SOR, MQRankSum, QUAL, ReadPosRankSum) and for short insertions and deletions (QD, FS, QUAL, ReadPosRankSum). Single nucleotide variants and short indels were annotated with ANNOVAR.

The following canonical transcripts were used in this work: *LDLR*: NM_000527.5, *APOB*: NM_000384.3, and *PCSK9*: NM_174936.4. For clinical interpretation, genetic variants with frequencies in the gnomAD database of < 0.5% or missing in the gnomAD were selected. Evaluation of the pathogenicity of the variants was carried out in accordance with the recommendations of the American College of Medical Genetics and Genomics (ACMG) with modifications [14]. All variants were analyzed for their presence in the databases (LOVD, ClinVar, and HGMD) [15,16]. A positive genetic diagnosis of FH was indicated by the presence of at least one pathogenic or likely pathogenic on one allele for the candidate gene. All the variants found in genes were confirmed by Sanger sequencing.

### 2.3. Statistical Analysis

Statistical analysis was conducted with Statistica software v8.0 (Statsoft Inc., Minneapolis, MN, USA) The data below are presented as a median (25th–75th percentile). A *p*-value of less than 0.05 was considered to be statistically significant. The *p*-values for quantitative parameters were calculated using a nonparametric Mann–Whitney test. The *p*-values for quality parameters were calculated using Yates corrected χ^2^ test. If a sample size was less than five, the two-tailed Fisher exact test was used. We calculated the prevalence of HeFH by dividing the number of people with definite FH, probable FH, definite or probable FH into total sample size consecutively. The prevalence of each FH definition was worked out as a percentage for all participants. Differences in FH prevalence between regions and for genetically confirmed FH were compared with Fisher’s exact test.

## 3. Results

From 2013 to 2015, we completed the pilot phase of the study in three regions of Russia; the main phase of the study started in July 2017. Patients were recruited for the study from September 2017 to September 2019 in 10 regions of the Russian Federation. A total of 1721 participants who had LDL-C higher than 4.9 mmol/L, or who had LDL-C lower than 4.9 mmol/L but had statin therapy, were invited for additional examination by experts in FH. A total of 105 participants with definite or probable HeFH were identified and no patients with homozygous FH were identified (Table 1). The prevalence of HeFH was assessed in 18,142 participants from 11 regions, with an average response rate of 81.9%. Due to the low participant response rate in Samara and Voronezh regions (30% and 33%, respectively), the calculation of the prevalence of FH was not carried out in these regions.

The prevalence of patients with definite HeFH was 0.27% (95% CI: 0.19–0.34%), probable HeFH was 0.31% (95% CI: 0.23–0.40%), definite or probable HeFH combined was 0.58% (1 in 173) (95% CI: 0.48–0.69%). The maximum prevalence of FH was 1/111 in the Ivanovo region, where the response rate was the highest at 92%, and where genetic screening of all ESSE-RF study participants in the region revealed seven additional patients with FH. The minimum prevalence of FH was 1/309 in the Krasnoyarsk region where there was the lowest response rate, 58%. At the same time, there were no significant differences between regions in the prevalence of FH (*p* = 0.9) and genetically confirmed FH (*p* = 0.22). A separate comparison of the prevalence of genetically verified FH in the Ivanovo region versus the average prevalence of genetically verified FH in all other regions revealed that the groups significantly differ (*p* = 0.0045), which is explained by the genetic test performed in all study participants in the Ivanovo region.

Clinical characteristics of participants with HeFH are presented in Table 2. A total of 16.1% of patients with definite or probable HeFH had tendon xanthomas and 36.2% of patients had mutations in one of the three genes (*LDLR*, *APOB*, and *PCSK9*) (Table 3). A total of 45.6% of FH patients had CAD, and 15.6% of patients had myocardial infarction. Despite guideline recommendations for addition of non-statin therapy to maximally tolerated statin for HeFH patients not at LDL-C goal [9], we noted suboptimal intensification of lipid-lowering therapy between the original visit when participants were included in the ESSE-RF study and this study visit. Only 63% of the HeFH patients received statins, only one patient was treated with statin and PCSK9 inhibitor, and nobody received ezetimibe (Table 2). Only three HeFH patients reached the LDL-C goal based on 2019 ESC/EAS guidelines [9]. Only three HeFH patients reached the LDL-C goal. One patient with HeFH and CAD treated with atorvastatin 40 mg per day and evolocumab 140 mg once every two weeks had an LDL-C level of 0.59 mmol/L. Two patients with HeFH and without CAD using maximum dose of atorvastatin had an LDL-C level of less than 1.8 mmol/L. The result of the assessment based on 2018 AHA/ACC guidelines was slightly better; six HeFH patients reached the LDL-C goal [17].

## 4. Discussion

The findings suggest that HeFH may be encountered in approximately 1 in 173 people in Russia, which is significantly more than was shown in the last two meta-analyses of 2020 [6,7].

According to a systematic review and meta-analysis by Pengwei Hu et al., about 50 population and cohort studies of FH prevalence have been conducted in various regions. Information on baseline characteristics and results obtained for all these studies are presented in the review tables. In these studies, the diagnosis of FH was based on a genetic test or accepted clinical criteria: Dutch Lipid Clinic Network (DLCN), Make Early Diagnosis to Prevent Early Deaths (MEDPED), Simon Broome diagnostic criteria (SB), Japanese Atherosclerosis Society guidelines criteria, Canadian FH criteria, or modifications thereof; or using total cholesterol or LDL-C cutoff points, frequently with additional clinical criteria such as personal or family history. The most frequently used criteria were DLCN and genetic test [6]. Large European epidemiological studies in Denmark, France, and Poland, where the DLCN criteria were used, have obtained data similar to ours on the prevalence of FH. According to the CGP Study, the prevalence of definite or probable FH was 1/218 [5,6]. Following the results of the MONICA and MONALISA studies, the prevalence of definite or probable HeFH was 1/117 (0.85% (95% CI: 0.63–1.06)) [17]. According to the HAPIEE Study, the prevalence of definite or probable FH was 1/183 (0.55% (95% CI: 0.39–0.69)) [6].

Advantages of our work are the investigation of the 18,142 participants from the epidemiological study, face-to-face examination of the eligible participants by experts in FH, estimation of tendon xanthomata and corneal arcus presence, and inclusion of the genetic testing results in FH diagnosing. We analyzed the contribution of genetic testing and physical examination criteria to identify patients with FH and calculated the prevalence of the disease (Table 3 and Table 4). Taking into account the family and individual history of CAD and hypercholesterolemia, biochemical results of the level of LDL-C within the DLCN criteria allowed us to identify 77 participants with HeFH (73% of all identified participants with HeFH) and the HeFH prevalence was 1/236. Consideration of tendon xanthomas and corneal arcus, in addition to the above, made it possible to diagnose an additional nine participants with HeFH (8.6%) and the HeFH prevalence increased to 1/211. Taking into account genetic testing data, in addition to the above, made it possible to additionally identify 19 participants with HeFH (18%) and the prevalence increased to 1/173. The prevalence of genetically confirmed HeFH was 1/477, which is similar to previously obtained data [6]. At the same time, when all participants of the population study were screened in the Ivanovo region, the prevalence of genetically confirmed HeFH was higher and amounted to 1/188. Thus, the recording of data from physical examination and genetic testing allowed us to identify an additional 27% of patients with HeFH. However, another factor that can explain the high prevalence of HeFH in Russia may be a higher prevalence of premature CAD in Russia than in European countries [18,19], which in combination with polygenic hypercholesterolemia can be mistaken for HeFH. In our study, we can identify 27 patients with 6 points by criteria DLCN, which they received due to hypercholesterolemia and premature CAD (level of LDL (4.91–6.44 mmol/L) − 3 points + family history of premature CAD − 1 point + clinical history of premature CAD − 2 points). None of these patients had a causal mutation. Excluding these patients from the calculation lowers the prevalence of FH to 1/233.

It has been shown that patients with familial combined hyperlipidemia (FCH) or a genetically determined increase in Lp(a) levels can be misdiagnosed as patients with FH [9,20]. We used the combination of ApoB > 120 mg/dL and TGs > 1.5 mmol/L with a family history of premature CVD to identify individuals with probable FCH [9]. Unfortunately, the level of ApoB and Lp(a) was available only for 64 patients with FH from six regions (Vologda, Ivanovo, Saint Petersburg, Tomsk, Vladivostok, and Tyumen) and an additional analysis was performed only for them. Fifteen out of sixty-four patients with FH (23.4%) had probable FCH, and only one of them had genetically confirmed FH. The maximum Lp(a) level was 220 mg/dL, the minimum Lp(a) level was 1.3 mg/dL, the median of the Lp(a) level was 13.9 mg/dL (95% CI: 8.3–58). Nineteen patients with FH had Lp(a) levels above 30 mg/dL. Fourteen out of sixty-four participants (21.8%) with definite or probable FH were reclassified as possible FH after adjusting LDL-C concentration for the cholesterol content (30%) of Lp(a), and only one of them had genetically confirmed FH. A total of twenty-four out of sixty-four patients (37.5%) had at least one of these conditions and only two of them had genetically confirmed FH. The obtained data may explain the relatively low detection rate of genetically confirmed cases of FH in the main group, which was only 36.2%. Exclusion of patients with probable FCH and reclassified patients with possible FH allowed us to increase the detection rate of genetically confirmed cases of FH up to 57.5%.

Compared to other clinical criteria (i.e., SB, MEDPED, and American Heart Association (AHA) criteria), DLCN criteria have the best balance of sensitivity and specificity, but they diagnose young patients without CVD relatively poorly and may lead to overdiagnosis in the case of polygenic dyslipidemias [20,21,22]. It has been shown that FH patients with monogenic FH variants have greater risk of CVD than patients in whom no causative variant is identified [22,23]. In our study, we used several approaches to diagnose patients with genetically confirmed FH: DLCN criteria, LDL-C cutoff (≥6.45 mmol/L) and for the Ivanovo region, a genetic test was carried out for all participants in the ESSE-RF study. We compared these approaches for the Ivanovo region before and after the correction for Lp(a) and FCH (Table 5). Genetic testing of all the adult population has a sensitivity index 3–5 times higher than DLCN and LDL-C cutoff (≥6.45 mmol/L) approaches. This approach allowed us to identify an additional seven participants with genetically verified FH, who were mostly young and without CVD, and their LDL-C level was in the range of 4.1–6.35 mmol/L. Similar results were obtained in the article by A.V. Khera et al., in which genetic diagnosis was carried out in 20,485 participants from five prospective cohort studies and where only 25% of people with identified causal mutations had an LDL-C level of more than 4.9 mmol/L [23]. These data indicate that the criteria for genetic testing need to be broadened to increase sensitivity in the detection of new cases of FH.

Before the availability of statins, there were several studies reporting the frequency of CAD in FH. In the study of Slack et al. [24], the incidence of CAD by 50 years in FH men and women was 85.4% and 56.5%, respectively. In the work of Stone et al. [25], 52% and 32.8% of FH men and women, respectively, had CAD by 60 years of age. Prevalence of CAD in FH patients in our study was 45.6%. Considering the high HeFH prevalence in Russia and the fact that everybody with HeFH was newly diagnosed in our study, we conclude that FH is underdiagnosed in Russia. Regarding high prevalence of CAD in individuals with FH and the low percent of FH patients treated with statin and non-statin lipid-lowering therapy and only 3% of HeFH patients achieving the goal LDL-C level, we can also deduce that FH is undertreated in Russia. Achieving the LDL-C goal for high- and very-high-risk patients, and especially for FH patients, is a worldwide challenge. Patients with HeFH initially have higher levels of LDL-C, and the effectiveness of lipid-lowering therapy, on the contrary, is reduced. The 2019 ESC/EAS guideline update recommends even lower LDL-C goals for very-high-risk and high-risk patients, including FH patients [9]. The data of the global registry of patients with FH (FHSC Registry) showed that only 3% of patients with FH had LDL-C levels less than 1.8 mmol/L [26]. In our study also, only 3% of FH patients reached the LDL-C goal based on 2019 ESC/EAS guidelines, and only 6% of FH patients reached the LDL-C goal based on 2018 AHA/ACC guidelines. Despite guideline recommendations for addition of non-statin therapy to maximally tolerated statin for HeFH patients not at LDL-C goal, in our study nobody received ezetimibe, which is due to the absence of ezetimibe on the Russian list of subsidized drugs. Unlike ezetimibe, PCSK9 inhibitors are included on the list of subsidized drugs, but they are still little used, not only in Russia, but also in other European countries. In our study, about 1% of patients received PCSK9 inhibitors, which is very similar to the results obtained in the DA VINCI study, where only 1.2% of very-high-risk patients received PCSK9 inhibitors [27]. Thus, we can also deduce that FH is undertreated in Russia. The situation with the treatment of FH patients can be improved through the widespread introduction of a combination of statins with ezetimibe and/or PCSK9 inhibitors.

Our study had some limitations. We estimated the prevalence of FH not as part of the main visit of the ESSE-RF study but as part of an additional study, the visits of which took place from 2 to 7 years later. The response of patients in the regions depended on the time interval between visits (the longer the time, the lower the response). In two regions (Samara and Voronezh) the repeated response rate was very low (30 and 33%, respectively). In these two regions, the average period was the maximum and amounted to about 6 years, but this was also due to the technical aspects of conducting research in these two regions. Excluding these two regions, the response rate averaged 81.9%. Although blood samples were available for the whole ESSE-RF study, genetic testing, however, was carried out for all participants only from one region; in other regions, testing was carried out for selected groups.

The prevalence of HeFH in the 11 Russian Federation regions is 1 in 173, which indicates a high frequency of HeFH in Russia. Almost half of individuals with HeFH had CAD. A total of 63% of patients with definite or probable HeFH were on statins, only <1% were on non-statin therapy, and with respect to the level of control, only 3% of the patients reached the targeted LDL-C level. Underdiagnosis and undertreatment of FH in Russia underline the need for the intensification of FH detection with early and aggressive cholesterol-lowering treatment.

## Figures and Tables

**Table 1:** Number of study participants and prevalence of HeFH in selected regions of the Russian Federation.

Region	Years of Recruiting Participants in the ESSE-RF Study	Years of Survey in This Study	Number of Participants	Number of Persons with LDL-C Level > 4.9 mmol/L	Number of Persons with LDL-C Level 1.8–4.9 mmol and Statin Treatment	Number of Persons with Definite FH	Number of Persons with Probable FH	Number of Persons with Mutations of *LDLR*, *APOB*, or *PCSK9*	Number of Persons with Definite or Probable FH	The Prevalence of FH
Krasnoyarsk	2014	2018–2019	1543	89	65	2	3	1	5	1/309
Vologda	2013	2018–2019	1650	157	14	5	3	3	8	1/206
Ivanovo	2012	2017–2019	1883	148	76	11	6	10	17	1/111
Saint Petersburg	2012	2018–2019	1600	135	58	4	5	4	9	1/178
Orenburg	2013	2018–2019	1596	75	53	3	5	3	8	1/200
Tomsk	2013	2018–2019	1600	158	43	4	6	4	10	1/160
Omsk	2017	2019	1645	71	113	2	5	2	7	1/235
Petrozavodsk	2017	2019	1647	60	66	5	5	5	10	1/165
Samara	2012	2018	1600	38	65	NA	NA	NA	NA	NA
Voronezh	2012	2018	1592	159	69	NA	NA	NA	NA	NA
Total in 8 regions (excluded Samara and Voronezh)			13,164	893	488	36	38	32	74	1/179
Vladivostok	2014	2015–2016	1726	162	69	2	5	1	7	1/247
Tyumen	2012	2013–2014	1630	142	10	6	7	3	13	1/125
Kemerovo	2012	2014–2015	1622	138	71	4	7	2	11	1/147
3 pilot regions			4978	442	150	12	19	6	31	1/161
Total in 11 regions			18,142	1335	638	48	57	38	105	1/173

ESSE-RF, Epidemiology of Cardiovascular Risk Factors and Diseases in Regions of the Russian Federation Study; FH, familial hypercholesterolemia; HeFH, heterozygous familial hypercholesterolemia; LDL-C, low-density lipoprotein cholesterol; NA, not applicable.

**Table 2:** Clinical characteristics of patients with diagnosed definite or probable HeFH (*n* = 105).

Parameters	Baseline Characteristics (ESSE-RF Study Visit)	FH-ESSE-RF Study Visit
Age, years	55 (50–61)	59 (53–66)
Men, (%)	38	38
Xanthomas, (%)	NA	16.1
Mutation of *LDLR, APOB*, or *PCSK9* (%)	NA	36.2
CAD after examination in the FH-ESSE-RF study, (%)	NA	45.6
Myocardial infarction, (%)	NA	15.6
Age of CAD starting, years	NA	52 (48–55)
Total cholesterol, mmol/L	8.05 (6.85–8.99)	7.3 (5.8–8.6)
LDL-C, mmol/L	5.97 (4.82–6.78)	4.5 (3.1–5.8)
Triglycerides, mmol/L	1.55 (1.11–2.02)	1.69 (1.32–2.18)
HDL-C, mmol/L	1.41 (1.2–1.71)	1.37 (1.15–1.63)
Statins, %	35	63
Ezetimibe, %	0	0
PCSK9 inhibitors, %	NA	1
Patients with goal LDL-C level (based on 2019 ESC/EAS guidelines), (%)	0	3
Patients with goal LDL-C level (based on 2018 AHA/ACC guidelines), (%)	0	6

CAD, coronary artery disease; ESSE-RF, Epidemiology of Cardiovascular Risk Factors and Diseases in Regions of the Russian Federation Study; FH, familial hypercholesterolemia; HDL-C, high-density lipoprotein cholesterol; HeFH, heterozygous familial hypercholesterolemia; LDL-C, low-density lipoprotein cholesterol; NA, not applicable.

**Table 3:** Data on each patient with mutations of *LDLR*, *APOB*, and *PCSK9*.

Region	Patients ID	Gene	Exon	DNA Change	Protein Change	dbSNP ID	gnomAD MAF (v. 2.1.1)	ClinVar ID
Ivanovo	240440	*LDLR*	11	c.1661C > T	p.Ser554Leu	NA	NA	251960
Ivanovo	240518	*APOB*	26	c.10580G > A	p.Arg3527Gln	rs5742904	0.0002942	17890
Ivanovo	240533	*APOB*	26	c.10580G > A	p.Arg3527Gln	rs5742904	0.0002942	17890
Ivanovo	240548	*LDLR*	13	c.1955T > C	p.Met652Thr	rs875989936	0.000003977	226382
Ivanovo	240605	*LDLR*	10	c.1474G > A	p.Asp492Asn	rs373646964	0.00002386	161285
Ivanovo	240629	*PSCK9*	9	c.1399C > G	p.Pro467Ala	rs772677312	0.00002829	265944
Ivanovo	240706	*LDLR*	5	c.798T > A	p.Asp266Glu	rs139043155	0.00003535	161287
Ivanovo	240846	*APOB*	26	c.10580G > A	p.Arg3527Gln	rs5742904	0.0002942	17890
Ivanovo	241451	*APOB*	26	c.10580G > A	p.Arg3527Gln	rs5742904	0.0002942	17890
Ivanovo	243117	*APOB*	26	c.10580G > A	p.Arg3527Gln	rs5742904	0.0002942	17890
Kemerovo	320465	*LDLR*	8	c.1129dup	p.Cys377fs	NA	NA	998054
Kemerovo	321005	*LDLR*	5	c.768C > A	p.Asp256Glu	rs879254671	NA	438322
Krasnoyarsk	40134	*LDLR*	9	c.1202T > A	p.Leu401His	rs121908038	NA	3735
Omsk	520435	*LDLR*	4	c.420G > C	p.Glu140Asp	rs879254520	NA	251216
Omsk	520819	*LDLR*	6;7	c.829G > A; c.976T > C	p.Glu277Lys; p.Ser326Pro	rs148698650; NA	0.0005056;	183097; 998053
Orenburg	530016	*LDLR*	4	c.343C > T	p.Arg115Cys	rs774723292	0.00002792	251162
Orenburg	530104	*LDLR*	12	c.1775G > A	p.Gly592Glu	rs137929307	0.00005656	161271
Orenburg	530905	*LDLR*	10	c.1502C > T	p.Ala501Val	rs755667663	0.000007954	251874
Petrozavodsk	860148	*LDLR*	7	c.1027G > A	p.Gly343Ser	rs730882096	0.00002832	183106
Petrozavodsk	860213	*LDLR*	9	c.1202T > A	p.Leu401His	rs121908038	NA	3735
Petrozavodsk	861317	*LDLR*	12	c.1784G > A	p.Arg595Gln	rs201102492	0.00003889	183126
Petrozavodsk	861359	*LDLR*	12	c.1784G > A	p.Arg595Gln	rs201102492	0.00003889	183126
Petrozavodsk	861627	*LDLR*	7	c.986G > A	p.Cys329Tyr	rs761954844	0.00002479	226344
Saint Petersburg	400857	*APOB*	26	c.10580G > A	p.Arg3527Gln	rs5742904	0.0002942	17890
Saint Petersburg	400882	*LDLR*	12	c.1750T > C	p.Ser584Pro	rs879255010		252015
Saint Petersburg	401046	*LDLR*	7	c.1048C > T	p.Arg350Ter	rs769737896	0.000007977	226342
Saint Petersburg	401056	*LDLR*	14	c.2001_2002delTG	p.Cys667_Glu668delinsTer	rs1600743301	NA	630543
Tomsk	690176	*LDLR*	12	c.1775G > A	p.Gly592Glu	rs137929307	0.00005656	161271
Tomsk	690307	*LDLR*	12	c.1747C > T	p.His583Tyr	rs730882109	0.0001025	200921
Tomsk	690427	*LDLR*	4	c.682G > A	p.Glu228Lys	rs121908029	0.00001614	3691
Tomsk	690787	*LDLR*	6	c.905G > T	p.Cys302Phe	rs879254715	NA	430768
Tyumen	710406	*LDLR*	12	c.1775G > A	p.Gly592Glu	rs137929307	0.00005656	161271
Tyumen	710818	*LDLR*	9	c.1202T > A	p.Leu401His	rs121908038	NA	3735
Tyumen	711388	*APOB*	26	c.10580G > A	p.Arg3527Gln	rs5742904	0.0002942	17890
Vladivostok	50260	*LDLR*	9	c.1202T > A	p.Leu401His	rs121908038	NA	3735
Vologda	190019	*APOB*	26	c.10580G > A	p.Arg3527Gln	rs5742904	0.0002942	17890
Vologda	191072	*LDLR*	12	c.1775G > A	p.Gly592Glu	rs137929307	0.00005656	161271
Vologda	191424	*LDLR*	9	c.1327T > C	p.Trp443Arg	rs773566855	0.000003980	NA

MAF, minor allele frequency; NA, not applicable.

**Table 4:** Prevalence of HeFH according to the different combinations of DLCN criteria.

Diagnostic Criteria	Number of Patients	Prevalence of FH in the Population According to the Diagnostic Criteria	Prevalence of FH in the Population by the Sum of the Criteria			
DLCN criteria without physical examination criterion (tendon xanthomas and/or corneal arcus) and results of genetic testing	77	1/236	1/236	1/211	1/173	
3 DLCN criteria: level of LDL (3 point) + family history of premature CAD (1 point) + clinical history of premature CAD (2 point)	27	1/789				
Other combinations of DLCN criteria	50	1/363				1/233
The tendon xanthomas or corneal arcus were necessary for the diagnosis of HeFH	9					
The genetic test was necessary for the diagnosis of HeFH	19					
Mutation of *LDLR*, *APOB*, or *PCSK9*	38	1/477				

CAD, coronary artery disease; DLCN, Dutch Lipid Clinic Network; FH, familial hypercholesterolemia; HeFH, heterozygous familial hypercholesterolemia; LDL, low-density lipoprotein; N/A, not applicable.

**Table 5:** Sensitivity, specificity, positive and negative predictive value, and Youden index of diagnostic test for FH.

	Sensitivity, (95% CI)	Specificity, (95% CI)	PPV, (95% CI)	NPV, (95% CI)	Youden Index
DLCN	20.0 (2.5–55.6)	99.6 (99.2–99.8)	22.2 (2.8–60.0)	99.6 (99.2–99.8)	0.196
LDL-C	30.0 (6.7–65.2)	99.4 (99.0–99.7)	21.4 (4.7–50.8)	99.6 (99.2–99.8)	0.294
DLCN corrected on Lp(a) and FCH	20.0 (2.5–55.6)	99.8 (99.5–100)	40.0 (5.3–85.3)	99.6 (99.2–99.8)	0.198
LDL-C corrected on Lp(a) and FCH	30.0 (6.7–65.2)	99.6 (99.2–99.8)	30.0 (6.7–65.2)	99.6 (99.2–99.8)	0.296

DLCN, Dutch lipid clinic network; FCH, familial combined hyperlipidemia; FH, familial hypercholesterolemia; LDL-C, low-density lipoprotein cholesterol; NPV, negative predictive value; PPV, positive predictive value.

## Data Availability

The datasets used and/or analyzed during the current study are available from the corresponding author on reasonable request.

## Appendix A

Principal investigator: S.A. Boytsov.

Study coordinator: A.N. Meshkov.

Central research team:

Federal State Institution “National Medical Research Center for Therapy and Preventive Medicine” of the Ministry of Healthcare of the Russian Federation, Moscow (Alexey N. Meshkov, Alexandra I. Ershova, Anna V. Kiseleva, Stepan A. Smetnev, Victoria I Makogonenko, Anastasia V. Blokhina, Alena S. Limonova, Evgeniia A. Sotnikova, Olga P. Skirko, Olga V. Kurilova, Anastasia A. Zharikova, Marina V. Klimushina, Mikhail G. Divashuk, Irina A. Efimova, Maria S. Pokrovskaya, Yuri V. Vyatkin, Vasily E. Ramensky, Vladimir A. Kutsenko, Svetlana A. Shalnova, Anna V. Kontsevaya, Oxana M. Drapkina).

Centers and Investigators:

Ivanovo: Ivanovo Regional Cardiology Dispensary (Elena A. Shutemova, Olga A. Belova, Elena S. Stroykova, Yulia Y. Kenina); Kemerovo: Research Institute for Complex Issues of Cardiovascular Diseases (Olga L. Barbarash, Elena V. Indukaeva, Yana V. Danilchenko, Olga K. Kuzmina); Krasnoyarsk: Krasnoyarsk State Medical University named after prof. Voino-Yasenetsky of the Ministry of Healthcare of the Russian Federation (Yury I. Grinshtein, Vladimir V. Shabalin, Alexandra A. Kosinova, Ruslan R. Ruf, Irina V. Filonenko); Omsk: Federal State Budgetary Educational Institution for Higher Education “Omsk State Medical University” of the Ministry of Healthcare of the Russian Federation (Inna A. Viktorova, Maria A. Livzan, Yulia N. Zakharevich, Irina V Salova, Irina I Zagoruy); Samara: Samara Regional Cardiology Dispensary (Dmitry V. Duplyakov, Raisa R. Kudraleeva, Natalia A. Cherepanova); Orenburg: Federal State Budget Educational Institution for Higher Education “Orenburg State Medical University” of Ministry for Healthcare of Russian Federation (Roman A. Libis, Irina R. Basyrova); Petrozavodsk: City Polyclinic №1 (Natalia N. Prishchepa, Ekaterina N. Alekseeva, Inga S. Skopets); Saint Petersburg: Federal State Institution “Almazov National Medical Research Center” of the Ministry of Healthcare of the Russian Federation (Oksana P. Rotar, Asiat S. Alieva, Anastasia M. Erina, Maria A. Boyarinova); Tomsk: Cardiology Research Institute, Tomsk National Research Medical Center, Russian Academy of Sciences (Victoria N. Serebryakova, Irina A. Trubacheva, Maria A. Kuzmichkina, Anna A. Brodskaya); Tyumen: Tyumen State Medical Academy (Irina V. Medvedeva, Marina A. Storozhok, Alexey Y. Efanov); Vladivostok: Pacific State Medical University (Vera A. Nevzorova, Dmitry Y. Bogdanov); Vologda: Vologda Research Center of the Russian Academy of Sciences (Olga N. Kalachikova, Mariya A. Gruzdeva), Vologda city polyclinic №1 (Irina V. Kosheleva, Galina V. Konovalova), Central district hospital of the city of Sokol (Galina N. Kotova); Voronezh: Voronezh State Medical University named after N.N. Burdenko (Tatiana M. Chernykh, Galina N. Furmenko, Andrey V. Redika, Vera V. Ovsyannikova).
